# Polarization-Addressable Optical Movement of Plasmonic Nanoparticles and Hotspot Spin Vortices

**DOI:** 10.3390/nano14100829

**Published:** 2024-05-09

**Authors:** Sergio Balestrieri, Silvia Romano, Mario Iodice, Giuseppe Coppola, Gianluigi Zito

**Affiliations:** National Research Council, Institute of Applied Sciences and Intelligent Systems, Via Pietro Castellino 111, 80131 Napoli, Italy; silvia.romano@na.isasi.cnr.it (S.R.); mario.iodice@na.isasi.cnr.it (M.I.); giuseppe.coppola@na.isasi.cnr.it (G.C.)

**Keywords:** plasmonics, spin–orbit coupling, nanophotonics

## Abstract

Spin–orbit coupling in nanoscale optical fields leads to the emergence of a nontrivial spin angular momentum component, transverse to the orbital momentum. In this study, we initially investigate how this spin–orbit coupling effect influences the dynamics in gold monomers. We observe that localized surface plasmon resonance induces self-generated transverse spin, affecting the trajectory of the nanoparticles as a function of the incident polarization. Furthermore, we investigate the spin–orbit coupling in gold dimers. The resonant spin momentum distribution is characterized by the unique formation of vortex and anti-vortex spin angular momentum pairs on opposite surfaces of the nanoparticles, also affecting the particle motion. These findings hold promise for various fields, particularly for the precision control in the development of plasmonic thrusters and the development of metasurfaces and other helicity-controlled system aspects. They offer a method for the development of novel systems and applications in the realm of spin optics.

## 1. Introduction

In nanophotonic systems, a variety of extraordinary properties and phenomena, leading to key technological innovations, descend from the coupling of photon spin angular momentum (SAM) with its spatial degrees of freedom [1,2]. Spin–orbit coupling can be observed in basic optical phenomena, such as the light propagation in anisotropic media [3,4,5,6,7], nonparaxial optics [8,9,10], and reflection/transmission at dielectric interfaces, manifesting the spin Hall effect of light [11,12]. It has been theoretically and experimentally demonstrated that electromagnetic waves also reveal a quantum spin Hall effect in analogy with the electronic effect in topological insulators [13]. In particular, the spin vector of the light has a nontrivial transverse component (tSAM) with respect to the direction of propagation, whose orientation locks the direction of the light wave [14,15]. The helical two-way edge states in Maxwell’s equations are the evanescent waves. The transverse magnetic propagating surface plasmon polaritons were the first evanescent waves used to demonstrate the spin–momentum locking [16,17]. As a result, the spin-directive coupling of light can be induced in arbitrary photonic systems sustaining evanescent-wave coupling because of the locking mechanism [16,18,19]. Evanescent waves can also exert forces and moments produced by tSAM acting on particles [14,20,21]. Numerous applications have been derived, such as active selection [22] and chiral manipulation [23,24] via metasurfaces, the analysis of biologic complexes and structures [25], and the separation of chiral enantiomers with lateral forces induced by spin–momentum coupling [26].

In gold nanoparticles, the localized surface plasmon resonance (LSPR) evanescent wave induces nonzero tSAM along the sphere surface, affecting the forces and torques induced by the electromagnetic field [27]. However, no investigation has been reported about the influence of tSAM and Belinfante’s spin momentum on the dynamics of the excitation source itself, i.e., the LSRP gold nanoparticle. While a single plasmonic nanoparticle is the geometry model for LSPR studies, another important resonant plasmonic configuration that induces enhanced local electric fields consists of a dimer of coupled nanoparticles owing to the gap hotspot; however, no investigation has been reported on this topic yet.

In our work, we first explore the mechanical action exerted on a plasmonic nanoparticle in a resonance regime as a result of Belinfante’s spin momentum [14,28]. We show that the trajectory of a gold monomer can be addressed by the polarization of the incident beam. This polarization-dependent variation involves many applications regarding the control of plasmonic particles, such as in optical tweezers and metasurfaces [29,30], and also in the plasmonic propulsion of nanoparticles [31,32,33]. Secondly, we study the behavior of the spin angular momentum in the case of a gold dimer. It is well-known that plasmonic nanostructures like dimers exhibit peculiar local field characteristics because of resonance hybridization and hotspot formation [34,35]. We report the formation of unique and topologically nontrivial textures of SAM densities in the gap hotspot. A pair of an SAM vortex and anti-vortex is formed on the opposite coupled nanoparticles. The formation and spatial separation of the quantized topological charges associated with such singular SAM density patterns have relevance for fundamental physics and spin optics.

## 2. Materials and Methods

The finite element method (FEM) model, simulated with COMSOL Multiphysics software v.6.2, used to obtain the results described above consists of a sphere of radius 10 nm positioned at the origin of the reference system, together with a second sphere of radius 400 nm, also at the origin of the reference system, enclosing the first sphere. The first sphere is of gold material, modeled with Johnson and Cristy model [36], representing the LSP source, whereas the second sphere, made of air material, serves as the surrounding environment; the calculation domain is spherical, rather than the typical cubic configuration used in this type of numerical simulations, in order to align the symmetrical properties of the field induced by the scatterer and with the radiative Sommerfeld conditions [37]. The light is constructed using a background wave defined by the following equation:

(1) $$E_{i}=E_{0}a_{j}e^{i(kz-\phi_{j})}\;j=1,2,3$$

The incident power ($E_{0}$) is set at $10^{7}$ V/m, and the polarization of the system ($a_{j}$) and the phase on the beam ($\phi_{j}$) can be controlled so that both linear and circular polarizations can be set. Conditions were then added to the scattering edge along with a perfectly matched layer that surrounds the air sphere to avoid multiple field reflection effects. We used a Livelink algorithm for force evaluation using both COMSOL Multiphysics v.6.2 and MATLAB software v.2023b. In particular, the code follows these steps:

1. Force evaluation, via Maxwell Tensor, using COMSOL v.6.2 Simulation of the model;
2. Force result processed in MATLAB v.2023b and evaluation of dynamics result (speed and position) using the motion equation; the time step of the dynamics is imposed previously ($10^{-8}$ s);
3. The new position of the gold nanoparticle is added in the FEM model for a new COMSOL v.6.2 evaluation of the optical force;
4. The cycle is repeated.

In this model, the gold sphere and the substrate sphere are both translated by the Livelink algorithm, ensuring that the nanoparticle remains within the evaluation domain without the possibility of moving away. The dimer simulation consists of the same model, placing another sphere of radius 10 nm such that they were located at a distance of 0.5 nm from the center of the reference system, obtaining a gap of 1 nm. In the Livelink calculation for dimer motion, an incident beam with an intensity of $10^{4}$ V/m and an initial gap of 10 nm between two particles were employed to visualize the displacement. The motion of each nanoparticle was evaluated independently.

## 3. Results

Given an electromagnetic wave described by Maxwell’s equations and an object placed in the beam, it is possible to define a tensor containing all the moments per unit of time resulting from the interactions of the object with the electromagnetic field. This tensor, known as the Maxwell stress tensor, is defined as follows [38]:

(2) $$T_{ij}=\epsilon_{0}(E_{i}E_{j}-\delta_{ij}{\left|E\right|}^{2})+\left(1/\mu_{0}\right)(B_{i}B_{j}-\delta_{ij}{\left|B\right|}^{2})$$

with $E_{i}$ and $E_{j}$ electric field components, $B_{i}$ and $B_{j}$ magnetic induction components, |E| and |B| the electric and the magnetic induction field amplitude, $\epsilon_{0}$ and $\mu_{0}$ are, respectively, the dielectric permittivity and magnetic permeability in vacuum. Since the Maxwell tensor is inclusive of all moments, it is possible to extrapolate only the momentum induced by the electromagnetic field; this momentum p is a function of the Poynting vector [39] such that

(3) p=18πωkRe(E*×H)

where *k* is the wavenumber, and ω is the frequency of the incident radiation.

In 1939, Belinfante suggested that the momentum induced by the electromagnetic field has two components [28], a term called orbital momentum ($p_{O}$) and a term called spin momentum ($p_{s}$):

(4) $$p=p_{O}+p_{s}$$

Given an elliptically polarized plane wave, the orbital momentum generates gradient forces and an orbital angular momentum (OAM); the spin momentum otherwise is a quantity defined as virtual because it does not generate any force but induces spin angular momentum (SAM) in the function of the spin density s.

Given Equations (2) and (3), it is possible to describe the spin density, spin momentum, and orbital momentum as a function of the electromagnetic field such that

(5) s=116πωIm[E*×E+H*×H]ps=12∇×spO=116πωIm[E*·∇E+H*·∇H]

In an evanescent wave, **s** acquires the following form [14,27]:

(6) $$s=\frac{\tilde{w}}{\omega }\left(\sigma \frac{k}{k_{z}}\hat{z}+\frac{\kappa }{k_{z}}\hat{y}\right)$$

where $\tilde{w}=\frac{1}{8\pi }{\left|A\right|}^{2}e^{-2\kappa x}$, $k_{z}$ is the longitudinal wavenumber and $\kappa =\sqrt{(}k^{2}-k_{z}^{2})$ is the exponential decay rate. The second term in Equation (6) is the tSAM, which, if generated by an evanescent wave, is capable of altering the trajectory of nanoparticles. Thus, the nanoparticles are not only affected by $p_{O}$ but also by $p_{s}$ [14]. The optical forces applied on the nanoparticles are [38]

(7) $$\langle F\rangle =\int_{\delta V}\langle T\rangle \cdot n(r)dS$$

where the force and the Maxwell tensor (T see Equation (2)) are time-averaged and n(r) is the normal along the surface of the nanoparticle and δV is the boundary where the force is evaluated.

### 3.1. Trajectories of Gold Monomers

**Figure 1:**
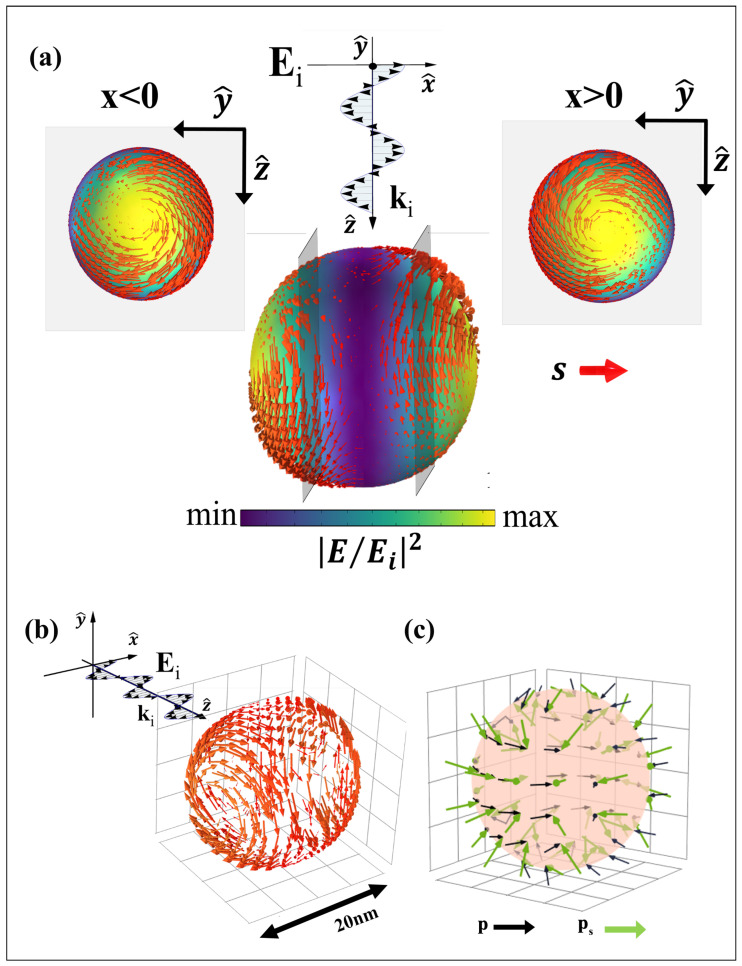
(**a**) Pseudocolor plot of the electric field amplitude normalized with the x-polarized incident field: the red arrow shows the spin distribution on the sphere and in the insets a 2D spin distribution on the *yz* section of the sphere. (**b**) 3D spin distribution along the nanoparticle surface. (**c**) Spin momentum distribution (green arrow) and momentum of the Poynting vector (black arrow) along the nanoparticle surface.

The evanescent waves generate a transverse spin momentum in addition to the usual orbital momentum resulting from the incoming beam. The resulting momentum acts on a probe particle, a phenomenon previously discussed in [27], observing a trajectory shift. Our investigation, unlike in [27], demonstrates that not only does the probe particle in the vicinity of the evanescent waves undergo trajectory modifications but the source itself, the resonant gold nanoparticle, undergoes the influence of their tSAM. The simulation model, designed with COMSOL Multiphysics v.6.2, involves a 10 nm radius gold sphere immersed in air and illuminated by a polarized plane wave of 530 nm in wavelength: the resonant wavelength for the plasmonic gold sphere. Figure 1 illustrates the resulting spin distribution (s) and spin momentum distribution ($p_{s}$) within the plasmon monomer, with the incident wave directed along the *z* axis and polarized along the *x* axis. The electric field distribution in this configuration exhibits an enhancement in the poles along the *x* axis (Figure 1a). As for Equation (5), the spin density (s) peak is at the same position of the electric field maximum, directed tangentially to the *yz* plane, resulting in vortex formations according to [40]. When observing the spin distribution along the spherical surface (Figure 1b), there are two distinct orientations of the s vector, symmetrical to the *yz* plane. In particular, the spin vector along the surface with x>0 exhibits an anti-clockwise vortex, while the corresponding one at x<0 displays a clockwise vortex. Considering the correlation between the spin vector and the electric field component, the symmetry of the *y* and *z* components of the electric field defines the orientation of the spin vector and its dependence on the hemisphere considered. This property is valid for any linear polarization, so it is possible to generalize the concept as follows: given a linearly polarized wave on a resonant gold nanosphere, s exhibits the maximum intensity in the poles along the axis of polarization, forming vortices with a direction anti-symmetrical to the plane orthogonal to the polarization direction considered.

**Figure 2:**
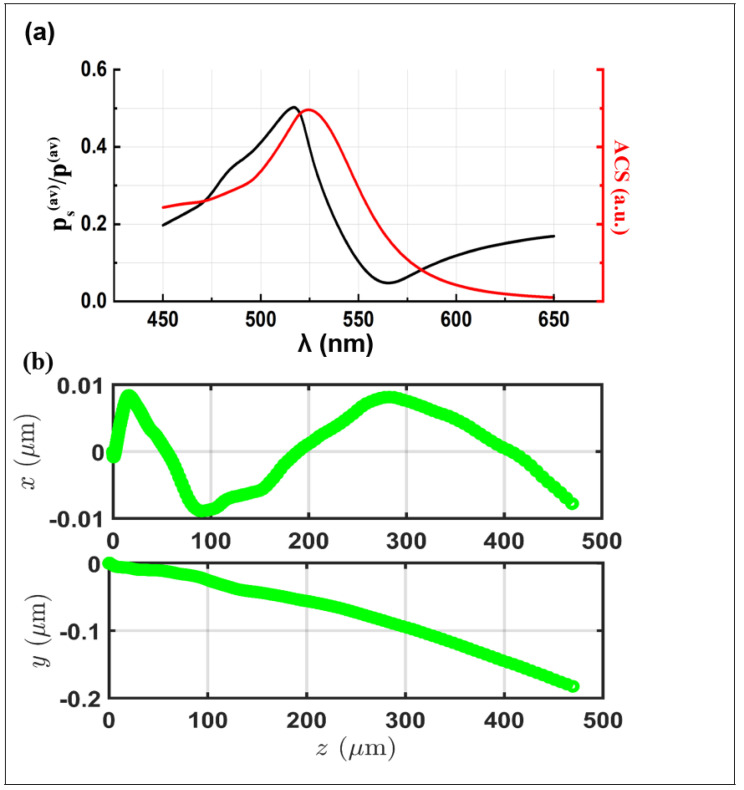
(**a**) Absorption plot (ACS) as a function of the incident wavelength (red line) and the ratio between the average spin momentum $\left(|p_{s}|\right)$ and the average momentum of the Poynting vector (|p|) as a function of the incident wave (λ) (black line). (**b**) The trajectory of a resonant gold nanoparticle under incident beam influence polarized along *x*.

The direction of the spin density (Figure 1a,b) is interconnected with the direction of the spin momentum $p_{s}$ (Figure 1c). Given the incident beam polarized along the *x* axis, the resulting spin momentum appears transverse to s, manifesting as a radial orientation towards the *yz* plane. The orientation of $p_{s}$ represents the behavior of s in the different surfaces of the gold nanoparticle, leading in two different directions symmetrical to the center of the sphere. The distribution of the spin momentum within the gold monomer (Figure 1c) exhibits a direct correlation with both the plasmonic resonance phenomena and the subsequent trajectory observed in the gold nanoparticle (Figure 2). Specifically, the ratio between the spin momentum amplitude (the Belinfante [28] component) and the Poynting vector in the function of the wavelength, depicted as the black line in Figure 2a, elucidates resonant peaks around 520/530 nm in which $p_{s}≃p/2$ (see Equation (4)). The examination of this plot alongside the absorption cross section (ACS) illustrated as the red line in Figure 2a accentuates a significant finding: the maximum spin momentum value coincides with the plasmonic resonance, i.e., the peak of the ACS curve, as revealed in [34].

**Figure 3:**
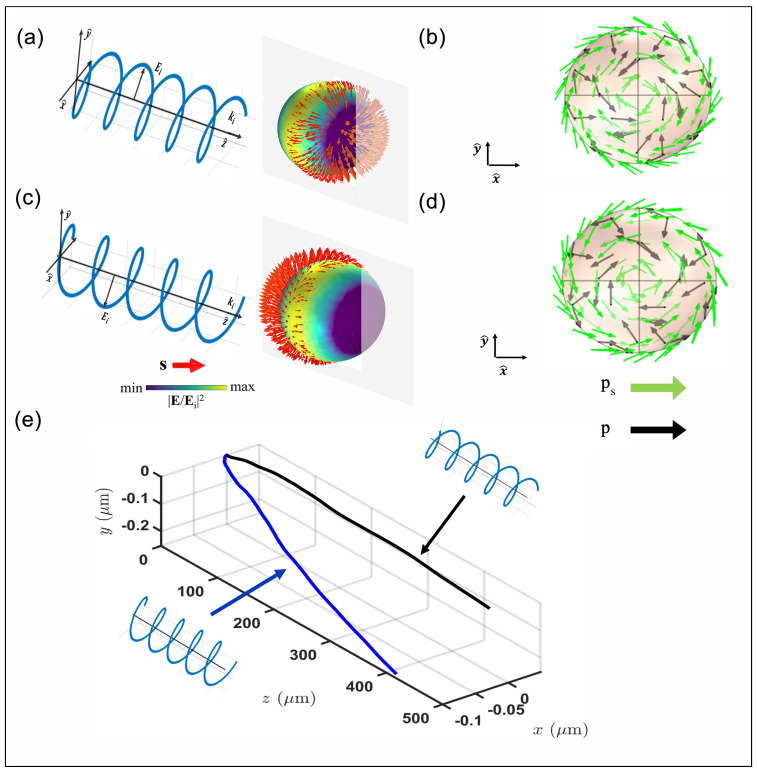
(**a**) Pseudocolor plot of the electric field amplitude normalized with the RCP-polarized incident field; the red arrow shows the spin distribution along the sphere. (**b**) Spin momentum (green arrow) and Poynting momentum distribution (black arrow) along the sphere surface with an RCP-incident polarization. (**c**) Pseudocolor plot of the electric field amplitude normalized with the LCP-polarized incident field; the red arrow shows the spin distribution along the sphere. (**d**) On the left: spin distribution along the sphere surface with an LCP-incident polarization (red arrow); on the right: spin momentum (green arrow) and Poynting momentum distribution (black arrow) along the sphere surface with an LCP-incident polarization. (**e**) Trajectory of a gold resonant nanoparticle under the influence of an RCP-polarized (black line) and LCP-polarized (blue line) beam.

This alignment supports the findings outlined in [6], underscoring the substantial impact of $p_{s}$, specifically at plasmonic resonant wavelengths. This influence directly contributes to altering the trajectory of the nanoparticle. Moreover, outside the resonant peak, the spin momentum has a negligible contribution to the Poynting vector. In this scenario, only the orbital momentum $p_{O}$ influences the system. The force evaluation and the dynamic behavior of the gold nanoparticle (Figure 2b) reflect the previous study of the spin momentum. The force is computed with the evaluation of the Maxwell tensor, as shown in Equation (7), deriving the dynamic parameters through the equation of motion. As a consequence of the tSAM and the $p_{s}$ amplitude in the resonant wavelength (Figure 2a), the gold nanoparticle has an additional displacement along the *y* axis in addition to the expected displacement along the *z* axis induced by the orbital momentum ($p_{O}$). The dynamic of the nanoparticles exhibits a polarization dependence driven by the distinct characteristics observed in the $p_{s}$ distribution and the electric field distribution within an LSPR gold monomer. Specifically, for each linear polarization, the electric field distribution exhibits a maximum on the poles placed along the polarization direction. Equation (5) ensures that the spin vector and the spin momentum have the maximum amplitude in the region of the peaks of the electric field, directing $p_{s}$ orthogonal to the polarization direction (akin to the probe particle scenario in [27]). Consequently, the trajectory of the nanoparticle diverges, consistently transverse to the incident direction and dependent on the polarization of the electric field. The investigation of the resonant gold nanoparticles exposed to circular polarization is illustrated in Figure 3. The figure shows the spin vector (s) and the associated momentum ($p_{s}$), as formulated in Equation (5), along the surface of the gold nanoparticle, and the impact of the tSAM on the trajectory of the nanoparticle.

Given a right circularly polarized (RCP) beam incident on the resonant gold particle (Figure 3a), the resultant electric field reaches the maximum intensity along the surfaces orthogonal to the axis of incidence of the beam (in our case, the *z* axis). Consequently, the spin density (s) exhibits significant amplitude concentrated within one hemisphere of the nanoparticle, breaking the axial symmetries observed in linear polarization (Figure 1a). In the *xy* plane, the spin density vector has a radial direction directed outward on the sphere surface (Figure 3a). The vector $p_{s}$ (Figure 3b) has an amplitude, according to Equation (5), mainly distributed in the same hemisphere of the spin density distribution but tangential to the surface of the sphere, forming a clockwise vortex. In the left circularly polarized (LCP) scenario (Figure 3c), both the spin vector (s) and the spin momentum vector ($p_{s}$) follow a similar pattern as observed in the RCP case. However, their distributions maintain equivalent amplitudes compared to the RCP spin density, although displaying an inverted orientation within the *xy* plane (Figure 3d). From these simulation outcomes, it is evident that alterations in the electric field vectors with varying beam helicity generate changes in the spin orientation and its associated moment along the nanoparticle surface. This behavior regarding the spin momentum significantly influences the dynamics of the gold nanoparticle (Figure 3e), remembering that, when the gold particle approaches the plasmon resonance, the Poynting momentum has a relevant Belinfante term (Figure 1c). Consequently, the contribution of $p_{s}$ generates dynamics distinct from those typically induced by the $p_{O}$ momentum. Specifically, there are two different force effects associated with the spin momentum:

1. A helicity-independent contribution, derived from the tSAM, affecting the trajectory variation along the y axis for both polarizations;
2. A helicity-dependent contribution prompting a trajectory shift along the *x* axis, the orientation being determined by the incident helicity.

The two trajectories form parabola branches with symmetry to the *z* axis. The nanoparticle selects one of the two trajectories depending on the helicity of the incident beam.

### 3.2. Spin Momentum in Plasmonic Dimer

**Figure 4:**
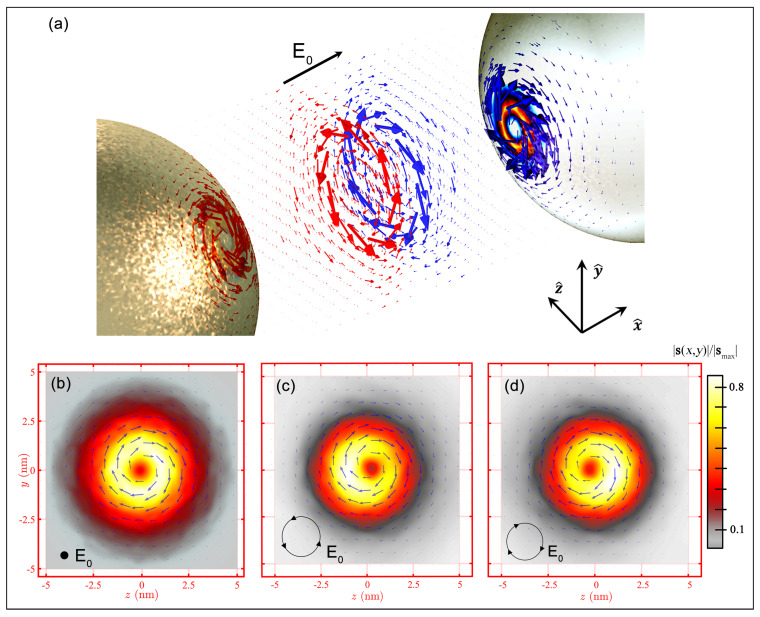
(**a**) Spin momentum distribution within the gap in a resonant gold dimer with incident polarization along *x*. Representation of the direction (illustrated by red arrows) and intensity (depicted through a pseudocolor plot) of the spin momentum distribution on a *yz* plane, positioned adjacent to the right particle in the gold dimer, under varying polarizations: (**b**) *x* polarization; (**c**) LCP polarization; (**d**) RCP polarization. The magnitude of the spin momentum is normalized to the maximum value (indicated by white in the color bar).

**Figure 5:**
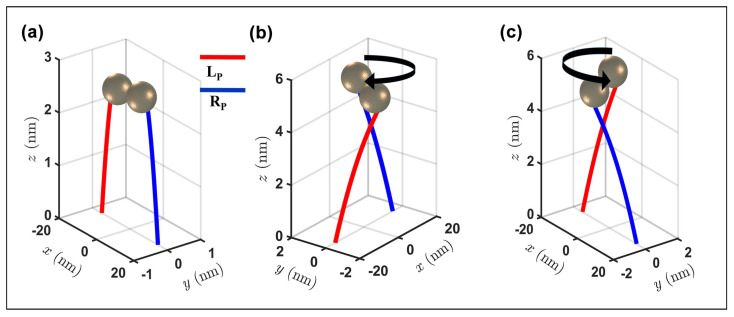
Trajectory of a gold resonant dimer (red line is the left particle; blue line is the right particle) with different incident polarization types: (**a**) x polarization; (**b**) RCP polarization; (**c**) LCP polarization. The sketch of the dimer indicates the point in space where the particles collide.

In the resonant gold monomer, the SAM behavior resulting from the evanescent surface plasmonic alters the nanoparticle trajectory, inducing a displacement dependent on the orientation of the Belinfante momentum ($p_{s}$). The spin–orbit effect in more intricate plasmonic configurations, the gold dimers, is now discussed. In the dimer, the resonant electric field shows an enhanced field localization within the gap between the gold particles [34], altering the characteristics of the spin vector associated with the system. The unique field distribution of the dimer generates a nontrivial topological configuration in the spin momentum. The spin distribution depends not only on the polarization of the incident beam, similarly to the SAM observed in the monomer, but also on the gap size between the two particles. Figure 4 shows the spin distribution (amplitude and orientation) of the dimer composed by two gold particles 10 nm in radius positioned parallel to the *x* axis, separated by a 1 nm gap, and exposed to incident polarized light, with a wavelength of 550 nm, directed along the *z* axis. In a dimer illuminated by an *x*-polarized incident beam, the field distribution, as reported in the literature [34], is enhanced within the gap between the two spheres. From Equation (5), the spin distribution has a higher intensity within the gap rather than along the surface of the nanoparticle, in contrast to the monomer behavior. Since the electric field components exhibit symmetry to the center of the gap, the spin vector aligns tangentially to the *yz* plane, orthogonal to the gap direction, forming a counterclockwise vortex near the left nanoparticle and a clockwise vortex near the right particle (Figure 4a). As one approaches the center of the dimer, where the electric field is most intense, the two vortices interact, altering their shapes until reaching the center of the gap, where the superposition effect is most pronounced, giving rise to a spin singularity. The amplitude distribution (Figure 4b) illustrates a symmetrical concentration of spin along the gap axis. As in Equation (5), the symmetric distribution of the electric field along the direction of the dimer gap corresponds to a similar characteristic exhibited by the spin. As the distance from the hotspot increases, the spin vector decreases in amplitude as a consequence of the direct correlation between the evanescent electric field component and the spin component. Similar to the behavior observed in the gold monomer, the presence and distribution of the spin vector on a specific surface are contingent upon the incident polarization. When illuminated with a *y*-polarized beam, i.e., transverse to the dimer gap direction, the electric field distribution is along the *xz* plane; thus, there is no excitation within the dimer gap. Due to the spin–electric field correlation, the spin vortex emerges along the *xz* surface of the nanoparticles, avoiding the dimer gap. As a result, in each nanoparticle, the spin distributions remain separated, precluding the formation of a spin singularity within the gap. Under illumination by a circularly polarized wave, the scattered electric field exhibits y component asymmetry dependent on the incident helicity. This asymmetry induces a helicity-dependent spin distribution manifested by the presence of a spin concentration in some areas of the gap. For LCP polarization (Figure 4c), this concentration appears in the area with z<0, while, for RCP polarization, it resides in the area with z>0 (Figure 4d). The behavior of the spin vector within the dimer has been previously investigated for various configurations, with a focus on the polarization-dependent effect. Figure 5 illustrates how the spin momentum, directly correlated with the spin vector, according to Equation (5), impacts the dynamics of the resonant gold dimer, resulting in displacements influenced by the incident helicity and polarization. In the linear polarization case, especially along the axis parallel to the dimer gap, an enhanced version of the tSAM generates a transverse moment $p_{s}^{z}$. This effect, evident in the dynamics of the dimer (Figure 5a), generates motion along the *z*-axis, elevating the dimer in addition to the expected motion along the gap axis, driven by the orbital momentum that attracts the two nanoparticles. For circularly polarized waves, the intrinsic helicity generates asymmetry in the *x* component of the spin, whose direction depends on the helicity; consequently, helicity-dependent momentum along the *y* direction is also formed. The dimer trajectory adopts a clockwise spiral shape in the RCP case (Figure 5b) and a counterclockwise pattern in the LCP case (Figure 5c).

**Figure 6:**
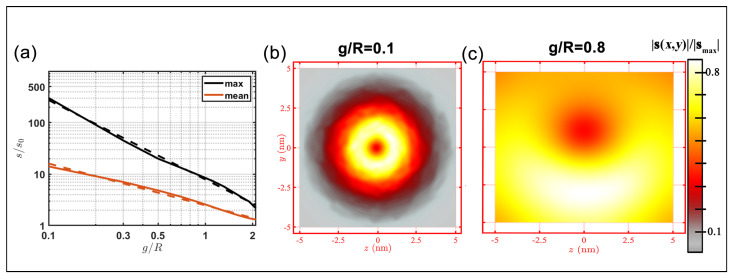
(**a**) Mean value (red line) and maximum value (black line) of the spin density in the gap of the dimer in the function of the parameter g/R. The spin value is normalized with the spin of a wave plane ($s_{0}$) circularly polarized, while the g/R parameter is the ratio between the gap of the dimer (*g*), normalized with the radius of the nanoparticle (*R*) value fixed at 10 nm; the dashed lines (red and black) represent the relative linear fits performed on the data. Pseudocolor plot of the spin intensity distribution, normalized with its maximum value for an *x*-polarized dimer configuration with two different g/R values: (**b**) g/R=0.1 and (**c**) g/R=0.8.

Figure 6 illustrates the analysis of the spin density within the dimer gap concerning the distance between nanoparticles while considering polarized incident light. In Figure 6a, s is normalized by the spin’s value in an incident plane wave with circular polarization ($s_{0}$). The parameter g/R represents the normalized distance between the nanoparticles (*g*), where the nanoparticle radius (*R*) used in this analysis is 10 nm. The mean (solid red line) and the maximum spin value (solid black line) exhibit a declining trend in the function of g/R. This decay can be attributed to their relation with the electric field, whose amplification decreases concerning the size of the gap. Given that the peak spin value aligns with the electric field hotspot, the analysis of the maximum aims to evaluate the spin behavior in the region where s is most intense relative to the dimer gap. Meanwhile, the average spin value considers the distribution of the vector across the entire gap and how it behaves concerning the inter-particle distance. A linear fit is employed for the plotted data, yielding trends described by log(y)=mlog(x)+A. The fit for the maximum value (dashed black line) yields *A* = 0.8951 and *m* = −1.5420, while, for the mean value (dashed red line), *A* = 0.3924 and *m* = −0.8126. When the g/R factor reaches 0.8, the amplitude distribution and orientation of the spin vector cease to be influenced by the coupling effect within the plasmonic dimer. At this point, the gold particles exhibit singular characteristics akin to those observed in the preceding paragraph, resembling the behavior of individual particles. Thus, in line with the characteristics of the LSPR concerning electric fields, the spin tends to decay as a function of the g/R ratio, reflecting the variation in the gap while holding the nanoparticle radius constant. In addition to the decrease in the amplitude of the spin (s), an alteration in the spin distribution within the dimer gap is observable with an *x*-polarized incident field (Figure 6c). Particularly, at a g/R factor nearing 0.8, an asymmetry emerges within the plane where z<0, resulting in an asymmetrical pattern concerning the *x* axis. This additional component, although present, is negligible for g/R < 0.8 due to the field amplification within the dimer (Figure 6b). It derives from the background field reflected from the gold nanoparticle, obtaining a shift in the electric field vector that acquires an angle concerning the axis parallel to the dimer gap.

## 4. Discussion

Analyzing the results, it is evident that, for LSP resonances in a gold nanoparticle, the trajectories are influenced by the spin momentum, resulting in shifts perpendicular to the direction of the incident beam. The literature often highlights how a probe particle placed in an evanescent wave (SPPs or LSPs) [1,27] presents a force contigent upon the SAM. However, in this paper, the system generates an evanescent wave, and, through the formation of spin momentum, it follows trajectories reliant on the polarization of the incident beam, owing to the induced plasmonic field. The ability to manipulate the position of a resonant system using a simple plane wave offers intriguing advantages, particularly in the plasmonic propulsion for the nanoparticle exit angle control [31,32,33]. In addition, functionalizing the plasmonic system holds promise in biosensing, drug delivery, and cancer therapy applications [25,26]. In the case of nanoparticle dimers, the electric field is spatially distributed within the dimer gap, and, consequently, the spin distribution is also present. The spin vector distribution in a dipole case tends to form vortices that overlap at the gap center, creating a spin singularity. The singularity formation, similarly to the monomer case, is influenced by the incident polarization and inter-nanoparticle distance. The presence of a singularity between a vortex and an anti-vortex confirms the existence of a topological charge in the plasmonic dimer system. The topological invariance, facilitated by the spin–orbit interaction (SOI), finds applications in spin optics, i.e., in the creation of metasurfaces [23,24], or in the semiconductor junctions controlled by the incident photon helicity [41].

**Figure 7:**
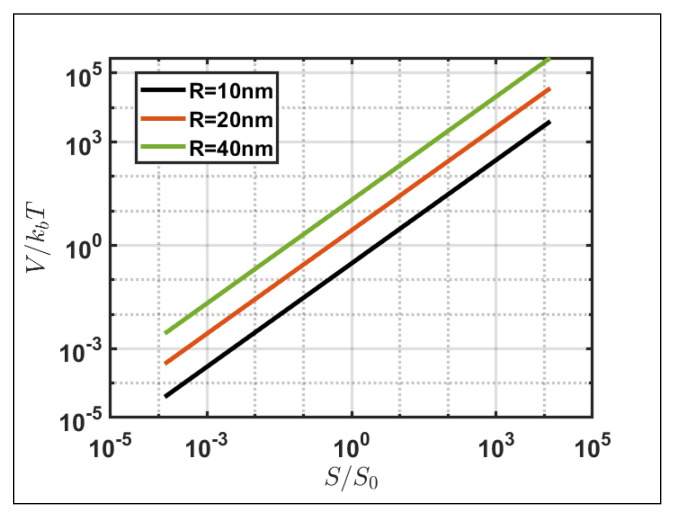
Logarithmic representation of nanoparticle electromagnetic energy normalized with $k_{B}T$, based on incident power density normalized with $S_{0}=1$ mW/μm^2^, parameterized for various nanoparticle radii.

For future prospects, the experimental implementation to analyze the spin behavior resulting from gold nanoparticles placed in water and a comparative study against the simulated analysis become imperative. First of all, the spin-induced trajectories are simulated assuming the surrounding environment to be air, while the nanoparticles will be placed in an aqueous solution for experimental verification. However, the analyses conducted on the motion of nanoparticles induced by the spin momentum are independent of the refractive index considered, excluding the increase in the frictional force caused by a denser medium but solely dependent on the incident polarization. Moreover, when assessing the experimental feasibility of the phenomenon in question, it is essential to account for the influence of Brownian motion, which introduces stochastic fluctuations in the trajectory of suspended gold nanoparticles [42,43]. Thus, a crucial aspect involves estimating the incident energy density necessary for the system to minimize the impact of the Brownian motion energy (k*_B_T*) on the trajectory of the nanoparticles. Figure 7 illustrates the electromagnetic energy normalized with $k_{B}T$ in the function of the incident power density, normalized at $S_{0}=$1 mW/μm^2^, a common value for microscopic element analysis. The plot considering different nanoparticle sizes reveals that, for power densities in the range of 10–100 mW/μm^2^, the electromagnetic energy exceeds the Brownian motion energy, affirming the experimental feasibility for the simulated spin–orbit phenomena. The required power density value depends on the nanoparticle size since the electromagnetic energy is related to the volume of the nanoparticle considered.

## 5. Conclusions

In this paper, we conducted a detailed analysis of spin (s) associated with momentum ($p_{s}$) and induced force behavior within a 10 nm gold particle forming an LSRP. We investigated the impact of an incident beam along the *z* axis, characterized by a wavelength of 530 nm with varying incident polarization, starting with a linear polarization and subsequently exploring LCP and RCP polarization. In addition, we examined the behavior of a gold dimer positioned along the x axis, separated by a gap of 1 nm, observing its behavior during the LSP resonance under identical incident beam conditions. From our investigation, we derived several key findings:

1. In the single-particle case, we observed spin distribution tangential to the plane orthogonal to the direction of polarization of the incident field, resulting in the formation of a vortex and an anti-vortex due to the symmetry of the system.
2. In a plasmonic dimer, the light forms a vortex and an anti-vortex within the gap only in some polarizations. The intensities depend on the gap and the polarization used; these two vortices collapse at the center, forming a spin singularity.
3. The dynamics of the resonant nanoparticles (single and dimer cases) exhibited deviations induced by the transverse spin momentum depending on the incident polarization.

The knowledge of the effects of spin on both the simple and dimer-configured nanoparticles has significant potential in exploring forces and torques. This offers advancement for the precision control in optical tweezers and development in plasmonic thrusters. Notably, the manifestation of chiral separation in nanoparticle trajectories may find applications in developing metasurfaces and intricate systems responsive to changes in beam helicity. The resonance features exhibited by plasmonic dimers open new avenues for studying complex plasmonic structures, such as particle chains or continuous resonant structures. These characteristics may benefit diverse applications, including nanoantennas and optical tweezers, by enabling dynamic control and substantial enhancements in their functionality.

## Data Availability

The raw data supporting the conclusions of this article will be made available by the authors on request.

## Abbreviations

The following abbreviations are used in this manuscript:

SAM	Spin angular momentum
tSAM	Transverse spin angular momentum
FEM	Finite element method
LSP	Localized surface plasmon
LSPR	Localized surface plasmon resonance
OAM	Orbital angular momentum
ACS	Absorption cross section
RCP	Right circular polarization
LCP	Left circular polarization
SPP	Surface plasmon polaritons
SOI	Spin–orbit interaction
